# Engineering Mesenchymal Stem Cells for Healthspan-Relevant Applications: Therapeutic Potential, Challenges, and Future Solutions

**DOI:** 10.3390/cells15161446

**Published:** 2026-08-11

**Authors:** Anne-Isabelle S. Reme, Mela Lew, Yulexi Y. Ortiz, Nga Le, Yan Li, Daniela Alexandra Ramos, Zhao-Jun Liu, Omaida C. Velazquez

**Affiliations:** 1 DeWitt Daughtry Family Department of Surgery, University of Miami Miller School of Medicine, Miami, FL 33136, USA; asr2250@miami.edu (A.-I.S.R.); mlew@brynmawr.edu (M.L.); yyo2@med.miami.edu (Y.Y.O.); ntl21@miami.edu (N.L.); yli3@med.miami.edu (Y.L.); dar420@miami.edu (D.A.R.); 2 Department of Psychology, Bryn Mawr College, Bryn Mawr, PA 19010, USA; 3 Department of Biochemistry & Molecular Biology, University of Miami Miller School of Medicine, Miami, FL 33136, USA; 4 Department of Radiology, University of Miami Miller School of Medicine, Miami, FL 33136, USA

**Keywords:** healthspan, aging, mesenchymal stem cells, cellular engineering, regenerative medicine, immunomodulation, senescence

## Abstract

**Highlights:**

**What are the main findings?**

- In various clinical trials, largely unmodified mesenchymal stem cell (MSC) therapies have shown limited and inconsistent benefits, highlighting the need for a shift toward MSC products engineered to target specific mechanisms and diseases.
- Targeted engineering, such as genetic modification, hematopoietic cell E-/L-selectin ligand (HCELL)/CD44 and E-selectin glycoengineering, hypoxic/cytokine preconditioning, biomaterial scaffolding, and MSC-derived extracellular vesicles, may address core limitations of unmodified MSCs by improving homing, persistence, immunomodulation, and resistance to senescence in preclinical models, which may in turn contribute to healthspan-relevant outcomes.

**What are the implications of the main findings?**

- Disease-matched and combinatorial engineering could establish MSCs as a flexible therapeutic platform for treating age-related diseases and potentially improving healthspan-related outcomes rather than lifespan alone.
- Clinical translation will depend on standardized manufacturing, validated potency and senescence resistance assays, long-term monitoring of tumorigenicity, and regulatory frameworks suited to aged recipients.

**Abstract:**

Engineered mesenchymal stem cells (MSCs) have emerged as promising therapeutic platforms for healthspan-relevant applications. As agents of tissue repair and modulators of biological aging, MSCs have been widely studied for their capacity to enhance regeneration, restore immune homeostasis, and reduce chronic inflammation associated with age-related decline. This review examines emerging bioengineering strategies designed to overcome key age-related limitations in MSC homing, survival, and paracrine signaling, which have historically constrained their in vivo efficacy. We discuss major engineering approaches, including genetic modification, surface engineering, metabolic reprogramming, and preconditioning, with particular attention to their contributions to longevity-focused applications. Preclinical studies have demonstrated that engineered MSCs and their extracellular vesicles (EVs) yield measurable improvements in therapeutic performance. Reported benefits include prolonged persistence in inflamed tissues, partial reversal of senescence-associated phenotypes, and modulation of pro-aging inflammatory pathways. While MSC-derived EVs may offer potential safety advantages and could reduce certain risks associated with live-cell administration, this remains to be confirmed in well-controlled clinical studies, and significant challenges persist in terms of manufacturing scalability, cargo consistency, and process standardization. The current literature, which is predominantly preclinical, supports the potential of engineered MSC platforms to improve healthspan-relevant outcomes; direct evidence of healthspan extension in humans is not yet available. However, successful clinical translation will require a standardized manufacturing process to ensure therapeutic safety, reproducibility, and efficacy in age-related conditions.

## 1. Introduction

Healthspan, defined as the period of life spent in good health without chronic disease or disability, is the central focus of aging research. Mesenchymal stem/stromal cells (MSCs) are multipotent stromal cells with immunomodulatory, regenerative, and trophic properties, making them promising candidates for therapeutic approaches that target age-related pathologies and may contribute to healthspan [1,2]. These properties have attracted substantial attention in anti-aging research, with evidence showing that MSCs regulate oxidative stress, attenuate endothelial cell senescence, defined as the irreversible arrest of cell division that accumulates in aging tissues, and promote angiogenesis [3].

However, the therapeutic efficacy of wild-type MSCs is limited by poor engraftment, low survival in harsh microenvironments, and reduced functional potency; the last of these is particularly evident when cells are derived from older donors [4,5,6]. Multiple human donor studies over the past decade have reported an age-associated decline in MSC potency, including reduced colony-forming unit fibroblast (CFU-F) frequency, slower proliferation, and diminished differentiation and paracrine capacity in both bone marrow and adipose tissue sources (Table 1). The relevance of healthspan to MSC biology is highlighted by the shared tissue-level mechanisms underlying many age-associated disorders, including chronic low-grade inflammation, vascular insufficiency, impaired proteostasis, mitochondrial dysfunction, stem/progenitor cell exhaustion (evidenced by the age-associated decline in MSC abundance and function, summarized in Table 1), extracellular matrix stiffening, and the accumulation of senescent cells [7,8,9].

Together, these changes impair tissue repair and injury responses by reducing stem cell self-renewal and differentiation while promoting a pro-inflammatory microenvironment through senescence-associated secretory phenotype (SASP) factors, namely, the pro-inflammatory cytokines, chemokines, and proteases released by senescent cells [15,16,17]. MSC-based interventions are therefore appealing because they may replenish depleted progenitor pools, attenuate inflammaging (chronic, low-grade inflammation that develops with age), and enhance endogenous repair mechanisms in age-related conditions [18,19]. Rather than functioning solely as replacement cells, MSCs act as dynamic, microenvironment-responsive signaling platforms that modulate multiple interconnected hallmarks of aging by adjusting their paracrine secretome in response to local inflammatory and senescent cues [3,16,17].

Nevertheless, MSCs remain vulnerable to replicative and stress-induced senescence, which often alters their secretory profiles and decreases their proliferative capacity [20]. A variety of engineering strategies have been developed to address these limitations and improve MSC survival, proliferation, and pro-regenerative capacity, thereby enhancing clinical performance [21,22]. These approaches include genetic and epigenetic modifications that increase the secretion of regenerative factors or strengthen resilience to adverse conditions, as well as preconditioning methods that enhance immunomodulatory activity [23,24,25]. For example, preconditioning MSCs with cytokines or hypoxic conditions improves their secretory profile and increases the production of anti-inflammatory cytokines [26].

The rationale for engineering MSCs is based on the well-documented age-related decline in stem cell potency, which compromises tissue homeostasis and limits the healing capacity of endogenous cell populations [27]. These limitations are intensified in pathological microenvironments. For example, ischemic tissues are characterized by hypoxia and nutrient deprivation, which reduce MSC survival; diabetic wounds contain high levels of advanced glycation end products, reactive oxygen species, proteases, and inflammatory cytokines, which impair proliferation and differentiation; and fibrotic tissues exhibit abnormal stiffness and disrupted integrin signaling, which promote maladaptive cell behavior [25,28,29,30].

Current strategies focus on optimizing MSC viability through targeted priming and genetic enhancement to counteract the SASP and functional exhaustion observed in vivo [31,32]. Senescent MSCs exhibit altered secretome compositions that can negatively impact tissue repair and homeostasis, necessitating strategies to counteract age-related functional decline [17]. This issue is significant because donor age is inversely correlated with MSC proliferation, differentiation, and immunoregulatory capacity, which may lead to detrimental “inflammaging” outcomes if aged MSCs are used therapeutically [25,33]. Therefore, investigating approaches that improve MSC function and mitigate the negative effects of cellular senescence is essential, especially in therapies targeting older populations [18].

This review examines advanced bioengineering strategies to optimize MSC characteristics for improved outcomes in age-related diseases, with an emphasis on overcoming age-associated functional decline and improving healthspan-related outcomes. Strategies to address donor age-dependent reductions in MSC functionality include deriving MSC-like cells from induced pluripotent stem cells, applying senolytics to remove senescent subpopulations, and enriching juvenile cell fractions through sorting, all of which aim to restore proliferative and immunomodulatory capacities [1,30,34]. In addition, biomaterial-based priming, pharmacological interventions, and customized culture conditions are being investigated to modulate MSC behavior and enhance their therapeutic properties [5,24,35,36,37].

Induced pluripotent stem cell-derived MSCs (iPSC-MSCs) offer a direct approach to bypass the donor-age restriction that affects primary MSC and mesenchymal progenitor cell (MPC) products. Since iPSCs can be clonally expanded and stored, iPSC-MSCs serve as an almost limitless, renewable cell source originating from a single, well-characterized clone. This approach effectively resolves issues related to scalability and lot-to-lot variability that occur with multiple isolations from older donors [38,39]. Reprogramming to pluripotency resets several aging features, such as telomere length, DNA-methylation age, mitochondrial function, and senescence markers. As a result, iPSC-MSCs derived from older individuals appear more youthful and less senescent than their original primary cells, while still maintaining standard MSC surface markers and the ability to differentiate into three lineages [40,41]. These features position iPSC-MSCs as a reliable, standardized platform for both therapeutic cell applications and consistent cell production for EV manufacturing [42].

Several challenges need to be addressed before iPSC-MSCs can be broadly used. Differentiation protocols from iPSCs to MSCs remain varied and lack full standardization, leading to inconsistencies in cell identity and potency. The presence of residual undifferentiated pluripotent cells poses a theoretical risk of teratoma formation, requiring sensitive detection methods and elimination strategies. Additionally, reprogramming and long-term culture may lead to genomic and epigenetic abnormalities, highlighting the need for thorough genetic stability testing [43,44]. Therefore, robust differentiation and purification workflows compatible with good manufacturing practice (GMP), clear release criteria, and comprehensive long-term safety data are essential [45]. Meeting these requirements would enable iPSC-MSCs to offer the convenience of an off-the-shelf, donor-age-independent source while providing the consistency essential for both cell- and EV-based products.

This review highlights three major strategies to improve MSC trafficking: increasing chemokine receptor expression, such as C-X-C chemokine receptor type 4 (CXCR4), through genetic approaches; using cytokine or hypoxia preconditioning to enhance migratory signaling; and engineering the cell surface with biomaterials to facilitate targeted endothelial adhesion [46,47,48]. Understanding these distinctions is essential for selecting the most appropriate strategy to address age-related declines in MSC homing efficiency, including reduced CXCR4 expression and impaired vascular cell adhesion molecule 1 (VCAM-1) mediated endothelial tethering in senescent microenvironments [16,30,49]. Careful consideration of the durability, safety, and compatibility of each approach with pathological conditions is necessary for optimal implementation.

## 2. Methods

This article is a narrative review rather than a systematic one, so no formal risk-of-bias assessment or meta-analysis was conducted. The literature search was conducted from February to July 2026, with the final structured searches performed in PubMed/MEDLINE on 28 July 2026, and in Europe PMC on 29 July 2026. Searches were limited to records published from 2000 onward. Four concept blocks were combined and searched in title/abstract fields with controlled vocabularies where available: (A) cell type, (B) engineering approaches, (C) aging-related terms, and (D) extracellular vesicles. Representative terms included mesenchymal stem/stromal cells and related progenitors; engineering and preconditioning strategies such as CRISPR, transduction, transfection, overexpression, scaffold, and biomaterial; aging terms such as healthspan, aging, senescence, frailty, and geroscience; and vesicle terms such as extracellular vesicles and exosomes. In PubMed, A AND B AND C yielded 1298 records (2 clinical trials); A AND D AND C yielded 663 records; A AND C yielded 5377 records (39 clinical trials), forming the pool for the trials summarized in Table 2. Equivalent Europe PMC searches (title-abstract, controlled vocab, no preprints) returned 1934, 1675, and 10,352 records, respectively.

Records were screened by title and abstract for relevance to engineered MSCs, MSC-derived extracellular vesicles, and aging- or healthspan-related outcomes. Additional references were identified from the reference lists of retrieved articles and from our prior knowledge of the field. Because this is a narrative review, final reference selection reflected our judgment rather than predefined eligibility criteria, and prioritized human clinical data over animal studies, animal studies over in vitro work, primary research reports over reviews and commentaries, and the most recent or largest study when several addressed the same point. Registered clinical trials were identified through ClinicalTrials.gov, and each record was verified against the registry. Literature on E-selectin-engineered MSCs was identified and included on the same basis as other engineering strategies; our related interests are declared in the Conflicts of Interest section.

Throughout this review, evidence is distinguished by study type, because the strength of support for healthspan-relevant claims varies substantially across designs. Four tiers are referenced: (i) in vitro studies, which can establish mechanism but not physiological benefit; (ii) animal aging models, including progeroid and naturally aged rodents and non-human primates, which can measure functional or biological aging endpoints but translate uncertainly to humans; (iii) disease-specific clinical trials, which in the MSC field predominantly report safety and organ- or disease-level functional outcomes rather than aging endpoints; and (iv) studies employing healthspan-relevant clinical endpoints, such as validated physical function measures, frailty indices, or biological aging biomarkers, which remain rare. Unless a study directly measured endpoints in the fourth category, this review uses the terms “healthspan-relevant” or “healthspan-related” outcomes rather than “healthspan extension.”

Cell types and product classes are distinguished as follows. “MSC” denotes culture-expanded mesenchymal stem or stromal cells meeting the International Society for Cell and Gene Therapy (ISCT) minimal criteria, irrespective of tissue source [62]. “Mesenchymal progenitor cell” (MPC) is retained where the original authors used that designation, including for pluripotent stem cell-derived progenitors that are not equivalent to primary MSCs. “iPSC-MSC” denotes MSC-like cells differentiated from induced pluripotent stem cells. “Immortalized producer cell” denotes a genetically stabilized line used as a manufacturing source for extracellular vesicles rather than as a therapeutic cell in its own right. For vesicle products, nomenclature follows the Minimal Information for Studies of Extracellular Vesicles guidelines (MISEV2023) [63]. The generic term “extracellular vesicle” is used throughout, and “exosome” appears only where it forms part of a registered trial record or is reproduced from a source that demonstrated endosomal origin.

## 3. Unmodified MSCs: Limitations and Challenges

The therapeutic utility of primary MSCs is often limited by poor engraftment and survival after systemic administration because culture-expanded cells lose critical migratory receptors, like CXCR4 [64]. Native MSCs rely on intrinsic homing receptors, stress-response pathways, and paracrine cues to reach target tissues. However, these mechanisms may be insufficient after transplantation into aged, damaged, or inflamed tissues. As a result, MSCs often show limited retention, short persistence, and inconsistent functional benefit despite favorable safety profiles [30,32,49]. Furthermore, these cells are rapidly cleared by the mononuclear phagocyte system and often become trapped in the pulmonary vasculature, limiting their distribution to distant target organs [65,66,67].

Disrupted homing mechanisms are especially important for healthspan-focused applications, where therapeutic cells must function within chronically diseased tissues characterized by persistent inflammation, oxidative stress, and fibrotic remodeling, which can worsen pulmonary entrapment and phagocyte clearance [30,68]. Donor-to-donor and tissue-source heterogeneity significantly increase variability in MSC potency due to differences in age, genetics, health status, and origin-specific secretory profiles, undermining batch consistency and clinical predictability [23,69,70]. Extensive ex vivo expansion required to achieve therapeutic cell numbers often leads to a loss of homing receptor expression and reduced metabolic plasticity [71].

Donor age, tissue source, expansion protocols, oxygen tension, and inflammatory stimuli strongly influence MSC potency by altering their proliferative capacity, homing receptor expression, and immunomodulatory functions [69,72,73]. These variations complicate the standardization of MSC products and challenge the development of reliable potency tests for preclinical studies [68,69,70]. In older donors, MSCs often experience accelerated senescence during expansion, leading to the loss of functional subpopulations and an increase in clones with reduced regenerative and immunomodulatory potential [16,25,33,70]. Serial passaging can further select for rapidly dividing but less effective clones, diminishing both heterogeneity and therapeutic versatility.

These age-related changes impair the ability to maintain a pro-regenerative secretome and immunomodulatory activity, as replicative senescence shifts the secretory profile toward proinflammatory SASP components that hinder tissue repair [16,17,74]. Unmodified MSCs exhibit inefficient homing and engraftment after systemic or local administration due to age- and expansion-related downregulation of chemokine receptors, such as CXCR4, and impaired VCAM- 1-mediated endothelial interactions. These factors lead to pulmonary entrapment and clearance by mononuclear phagocytes [30,46,48,49,68,69].

Following intravenous infusion, MSCs are rapidly sequestered in the pulmonary capillaries due to their size and reduced deformability, triggering an acute inflammatory response and facilitating phagocytic clearance within hours, thereby limiting their redistribution to sites of injury or inflammation [47,67,68]. Metabolic stress from pulmonary trapping further impairs the secretory function of aged MSCs, reducing the systemic availability of essential growth factors and cytokines required for immune modulation [75]. This impairment, combined with replicative senescence, diminishes sustained immunomodulatory interactions and exacerbates inflammaging in chronically inflamed environments [19,30,76].

These challenges hinder clinical translation by preventing standardized manufacturing, regulatory validation of potency, and safe systemic delivery in elderly patients. Donor heterogeneity and expansion-induced senescence increase the risk of inconsistent efficacy and may worsen inflammaging through impaired biodistribution and clearance [25,30,32,69]. Rigorous quality control and standardized isolation are essential for reducing this variability [77]. Conventional two-dimensional monolayer expansion exacerbates these issues by accelerating senescence, downregulating homing receptors such as CXCR4, and shifting the secretory profile toward proinflammatory mediators [69,70,74]. Emerging research suggests that preconditioning strategies, including hypoxic exposure or administration of specific growth factors, can enhance CXCR4 expression and improve MSC homing efficiency prior to clinical use [78].

## 4. Engineered MSCs: Mechanisms and Therapeutic Potential

**Figure 1:**
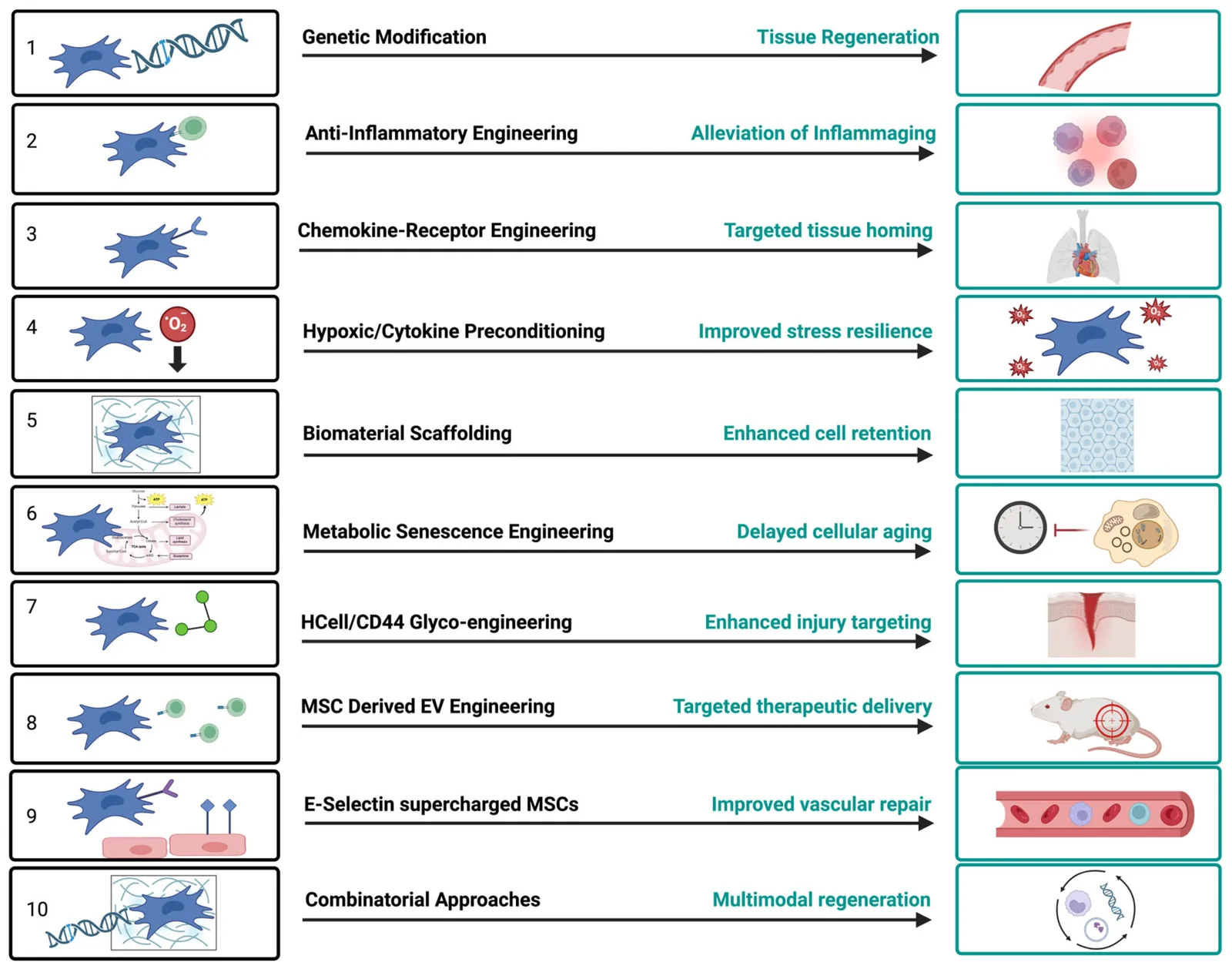
Engineering strategies to improve MSC performance for potential healthspan applications. Ten approaches: genetic modification [22], anti-inflammatory [25] and chemokine-receptor engineering [87], hypoxic/cytokine preconditioning [88], biomaterial scaffolding [28], metabolic senescence engineering [89], HCELL/CD44 glycoengineering [90], MSC-derived EV engineering [91], E-selectin engineering [60,92], and combinatorial methods [93]. Each is paired with its potential healthspan benefit, ranging from tissue regeneration and alleviation of inflammaging to delayed cellular aging and multimodal regeneration. This schematic is a conceptual summary of engineering strategies and their proposed benefits; it does not depict experimental results and should not be interpreted as evidence of efficacy. Created in BioRender. Reme, A. (2026) https://BioRender.com/hsqc8g7.

Multiple strategies have been designed to improve MSC performance, including genetic modification, microRNA treatment, preconditioning, and epigenetic reprogramming, each offering a distinct route to improved healthspan-related outcomes (Figure 1) [79]. These approaches aim to enhance key MSC characteristics, including homing ability, viability in challenging physiological environments, and targeted paracrine signaling. Genetic modification often uses viral vectors to introduce genes encoding growth factors or anti-apoptotic proteins, thereby increasing MSC survival and regenerative factor production [47,69]. Non-viral methods, such as transfection and electroporation, are also being refined to deliver therapeutic genes or regulatory RNAs, offering safer alternatives for clinical applications [80].

Surface modification strategies that use bioorthogonal chemistry or lipid-based coatings should be matched to the dominant biological challenge in each disease. For example, in ischemic cardiovascular conditions, these modifications can reduce pulmonary entrapment, whereas in inflammatory environments, they can decrease immune clearance. This approach may prolong systemic circulation and improve adhesion and function in target tissues [81,82,83]. In ischemic limb disease and chronic wounds, impaired stromal cell-derived factor 1 (SDF-1)/CXCR4 signaling limits cell recruitment, and poor endothelial adhesion delays angiogenesis and tissue repair. Therefore, disease-tailored surface modifications may enhance MSC retention and improve recovery [21,84].

The major challenge in immunosenescence and inflammatory disorders is rapid immune clearance and inflammation driven by insufficient receptor engagement. In these settings, surface modifications should support durable anti-inflammatory effects and improved communication with immune cells [25,30,76]. For neurodegenerative diseases and frailty syndromes, surface engineering can be designed to deliver neurotrophic factors, support mitochondrial function, and provide anti-senescent vesicles. These examples underscore the need for mechanism-driven, disease-specific MSC platforms rather than a one-size-fits-all approach [79,85,86].

### 4.1. Genetic, RNA, and Genome-Editing Strategies

In addition to genetic methods, small molecules and growth factors can modulate MSC behavior by activating signaling pathways that support proliferation and stress resistance [94]. Engineering the culture environment with defined physical, chemical, or biological cues can also guide MSC development and enhance their therapeutic efficacy [95]. Epigenetic reprogramming, including reversal of age-associated methylation patterns, may rejuvenate aged MSCs and improve their therapeutic utility [16].

CRISPR-Cas9 (clustered regularly interspaced short palindromic repeats and CRISPR-associated protein 9) enables precise genetic modifications that can enhance MSC survival, differentiation, and immunomodulatory functions [96]. For example, CRISPR/Cas9-mediated Keap1 knockout in adipose-derived MSCs activates the Nrf2 pathway, strengthening antioxidant defenses and improving cell survival [96,97]. Increasing the expression of C-X-C chemokine receptors types 1, 4, and 7 (CXCR1, CXCR4, CXCR7) can boost MSC migration and growth, leading to better outcomes in disease models [46]. Similarly, inserting the SV40 large T antigen (SV40T) into the Rosa26 locus has been shown to enhance the proliferative ability of bone marrow MSCs in vitro [98]. Since SV40T is a viral oncogene, this type of immortalization should be viewed as a research and cell-manufacturing tool rather than a proven or inherently safe clinical modification. These immortalized cells are typically not suitable for direct patient use; instead, they serve as producer cells for cell-free EV products, as explained below.

Engineering MSCs to express interleukin-10 (IL-10), hepatocyte growth factor (HGF), indoleamine 2,3-dioxygenase (IDO), Foxp3, or Bcl-2 can strengthen immunomodulation, reduce inflammation, and improve resistance to apoptosis [72,99]. Likewise, increasing expression of CXCR4, IL-10, brain-derived neurotrophic factor (BDNF), or interferon-β (IFN-β) using viral vectors or CRISPR/Cas9 can enhance MSC immune, antiviral, and neurotrophic functions [86]. Finally, post-transcriptional modification strategies may increase long-term MSC resistance to hostile microenvironments [100].

### 4.2. Preconditioning, Metabolic Reprogramming, and Biomaterial Priming

Pharmacological approaches using small molecules, cytokines, growth factors, hypoxia, extracellular matrix signals, and biomaterials can prime MSCs to maintain stronger secretory, migratory, and immunomodulatory traits under hostile conditions [24,25,101]. For example, diabetes impairs the reparative function of bone marrow-derived stem/progenitor cells, including MSCs (BM-MSCs). Ex vivo priming with SDF-1α can functionally activate these cells before transplantation. In the referenced study, SDF-1α-primed diabetic BM-MSCs significantly accelerated wound closure, increased neovascularization, and enhanced endothelial progenitor cell recruitment, while improving cell proliferation without reducing viability [102]. Mechanistically, SDF-1α priming upregulated pro-healing and proangiogenic mediators, including plasminogen and EphB4, and promoted adhesion-related pathways that may support endothelial progenitor cell interaction and vascular engraftment. Importantly, because SDF-1α exposure was performed ex vivo and cells were washed before implantation, this approach improved wound repair without increasing systemic SDF-1α levels [102]. These findings support SDF-1α preconditioning as a practical strategy to enhance the regenerative potency of MSCs/BM-MSCs for diabetic wound healing.

Exposure to hypoxia or inflammatory cytokines such as interferon-γ (IFN-γ) and tumor necrosis factor-α (TNF-α) can improve the MSC secretome and increase release of cytoprotective and immunomodulatory factors that are often reduced under standard culture conditions [73,88]. In addition, transforming growth factor-α (TGF-α) priming improves MSC survival in ischemic myocardium, demonstrating that targeted biochemical preconditioning can help overcome barriers to engraftment and expansion in vivo [103,104].

Metabolic reprogramming, such as increasing nicotinamide adenine dinucleotide (NAD+) levels or activating AMP-activated protein kinase (AMPK), improves MSC function in aged tissues by restoring mitochondrial health, balancing glycolysis, and increasing resistance to age-related decline [76,89]. These approaches can also precondition MSCs, reducing variability and improving clinical consistency [105,106]. Conversely, poorly controlled culture conditions can increase MSC heterogeneity, reduce immunomodulatory function, and decrease potency consistency [72,94]. To ensure reliable clinical outcomes, potency assays for engineered MSCs should incorporate mechanism-specific functional tests, including immunomodulatory secretome profiling, migration assays, and evaluation of key quality attributes [68,107,108].

### 4.3. Surface Engineering, Glycoengineering, and HCELL

Cell-surface engineering methods, including lipid-anchored palmitated protein G for antibody or ligand attachment, have been developed to address poor homing and tissue targeting in MSC therapy. These strategies display adhesion molecules that enhance selectin- or integrin-mediated binding, improving MSC trafficking to specific target sites or inflamed tissues [1,47]. Because MSCs commonly lack sufficient selectin ligands for effective capture under physiological flow, surface modifications such as enzymatic fucosylation of cell-surface glycans can increase vascular tethering and tissue entry without altering the cell’s internal program [1,109]. Glycoengineering provides a targeted strategy to increase selectin binding and improve MSC trafficking to inflamed tissues, thereby enhancing delivery efficiency [110].

Specialized scaffolds can also help maintain therapeutic activity and prolong MSC survival in inflamed environments [28]. Fucosylation of MSCs by converting CD44 to the hematopoietic cell E-/L-selectin ligand (HCELL) glycoform has been shown to enhance vascular adhesion and transendothelial migration into damaged tissues by activating integrin α4β1 [109]. This modification helps MSCs attach and roll along blood vessels under flow, overcoming their natural lack of E-selectin ligands [111,112]. Because native MSCs lack the sialofucosylated determinants required for E-selectin recognition, HCELL glycoengineering addresses poor bone targeting and limited vascular extravasation [90,113,114].

As a transient, non-genetic modification, this approach improves MSC homing to bone and inflamed sites while preserving cell viability and function and avoiding risks associated with permanent genetic alteration. These features have supported early clinical translation, including phase I trials in osteoporosis [54,55,110]. However, HCELL generation via glycosyltransferase-programmed stereosubstitution is limited to existing glycoproteins, such as CD44, and may unintentionally alter other surface molecules. It may also transiently shift the secretome toward anti-inflammatory effects, which could restrict broader therapeutic applications [1,90].

### 4.4. MSC-Derived Extracellular Vesicles as Cell-Free Engineering Platforms

MSC-derived extracellular vesicles (EVs) are promising cell-free engineering platforms that may overcome key limitations of cellular therapy, including tumorigenic risk and poor survival in hostile host microenvironments. These vesicles deliver concentrated bioactive proteins, lipids, and nucleic acids [91,115,116]. Their surface can be modified, or their cargo can be loaded with specific therapeutic agents to improve targeting and signaling, allowing them to mimic beneficial functions of parent MSCs while potentially reducing certain risks associated with systemic cell administration [117].

In animal aging models, vesicles from young or rejuvenated MSCs reduced senescence markers such as p16 and p21, decreased oxidative stress, and improved healthspan-related measures in Ercc1-deficient progeroid and naturally aged mice by delivering anti-senescence signals that restore stem cell function, while potentially avoiding some risks associated with direct cell therapy [118,119,120]. However, clinical translation of engineered EVs remains challenging due to batch variability across different cell sources and culture conditions, the lack of reliable large-scale isolation methods, and the absence of standardized potency assays to ensure consistent biodistribution and safety [91,115,121,122].

For therapeutic EV products, following community reporting frameworks is crucial for maintaining rigor and reproducibility. The MISEV2018 and MISEV2023 guidelines, along with the International Society for Extracellular Vesicles (ISEV)–ISCT position statement on MSC-derived EVs, establish the minimal standards for experimental design and reporting in the field [63,123,124]. In accordance with these guidelines, EV nomenclature requires a clear definition: use generic terms such as “extracellular vesicle” unless the subcellular origin is confirmed. Avoid unsubstantiated terms such as “exosome.” Reports should comprehensively detail the EV source, including cell type, donor and passage number, along with culture, preconditioning, and conditioned-medium harvesting conditions, as these parameters significantly impact EV identity and cargo. The method used for isolation and separation, such as differential ultracentrifugation, size-exclusion chromatography, tangential-flow filtration, or precipitation, must be described thoroughly enough to allow replication, noting that each technique produces preparations with different purities and possible co-isolated contaminants.

Characterization should integrate quantitative particle analysis, such as nanoparticle tracking analysis or tunable resistive pulse sensing, to determine particle concentration and size distribution, along with single-vesicle imaging techniques, such as electron or atomic force microscopy, to verify morphology. Identity confirmation should rely on markers described in MISEV2023 categories, including transmembrane or lipid-bound surface proteins (e.g., CD9, CD63, CD81), cytosolic proteins with membrane-binding ability (e.g., TSG101, syntenin, ALIX), and MSC-associated surface markers [63]. Furthermore, negative markers such as calnexin and, in serum cultures, albumin and lipoproteins, should be shown to be absent or depleted. Purity should be assessed quantitatively, for example, using particle-to-protein or particle-to-lipid ratios. Potency evaluation must rely on functional, mechanism-of-action-relevant assays rather than particle counts or surface markers alone, with clear acceptance criteria tied to the therapeutic objective. Finally, comprehensive and transparent reporting, including depositing methodological metadata in repositories such as EV-TRACK, will enhance reproducibility and enable better cross-study comparisons [63,125]. Applying these standards to MSC-EV products is essential for ensuring reliable potency comparisons, regulatory assessments, and successful clinical translation. Notably, EV safety cannot be broadly generalized. Since EV composition and related risks vary greatly depending on source cells, manufacturing techniques, and cargo, safety should be determined empirically for each manufacturing process and therapeutic use rather than assumed to be universal.

The strategy used to isolate and purify MSC-EV products is vital to their quality, but there is no single best method for all characteristics [126]. Differential ultracentrifugation has traditionally been the most common method as it is cost-effective and widely accessible. However, it scales poorly, provides limited throughput, and may co-pellet protein aggregates and lipoproteins, which can compromise vesicle integrity and reduce recovery [127]. Polymer precipitation methods are straightforward and yield high amounts but often co-isolate significant non-vesicular proteins and polymers, resulting in low purity, typically unsuitable for clinical applications. Size-exclusion chromatography (SEC) offers a gentle, consistent separation that maintains EV integrity and achieves higher purity than precipitation. However, SEC dilutes the sample, has limited volumetric capacity, and does not fully separate lipoproteins of similar size [128]. Tangential flow filtration (TFF) is highly scalable and compatible with closed systems, making it ideal for the large conditioned-medium volumes needed in manufacturing. It provides good recovery, but shear forces and membrane fouling need to be managed, and some protein and small-molecule contaminants may persist [128]. Immunoaffinity capture, which employs antibodies targeting tetraspanins or tissue-specific surface markers, provides the highest specificity and enables isolation of specific EV subpopulations. However, it is expensive, has low capacity, and is limited by elution conditions and potential antibody leaching, which restricts its scalability under GMP conditions [63]. In practice, orthogonal combinations, most often TFF for concentration and buffer exchange, followed by SEC for polishing, are increasingly employed to optimize scalability, purity, recovery, cost, co-isolated contaminants, and GMP compliance. The chosen process train should be justified for the specific product and finalized before critical manufacturing stages.

Robust quality control also demands the establishment of potency assays and batch-release criteria specific to each product. Since EV mechanisms are usually multifaceted, a quantitative bioassay relevant to the mechanism of action, such as an in vitro test measuring the intended effect like immunomodulation, angiogenesis, or target-cell signaling, should be paired with molecular surrogate markers (specific proteins or miRNA cargo) once these are proven to correlate with bioactivity [129]. Batch-release specifications should include identity markers specific to EVs, quantity measures like particle count and dose, purity indicators such as particle-to-protein or particle-to-lipid ratios with limits on residual host–cell protein and DNA, safety parameters including sterility, endotoxin levels, mycoplasma presence, and for engineered producer lines, residual cells, oncogenic nucleic acids, and vector components, all within predefined acceptance ranges [123,126]. Regulatory classification of EV products varies based on the source, degree of manipulation, and intended use. They may be regulated as biological products or as advanced therapy medicinal products (ATMPs) in the European Union. Some products or components could also fall under drug, device, or combination-product regulations. Additionally, unmodified EVs and gene-modified EVs may be classified differently [130,131]. Given this heterogeneity, it is recommended to engage early with the relevant regulatory authority and establish a clear product classification. This helps ensure that the characterization, potency, and release requirements are aligned with the appropriate regulatory pathway.

Its utility could be advanced through standardized proteomic analysis, functional assays, and quality attributes directly linked to treatment outcomes [115,123]. In animal aging models, engineered MSCs, including senescence-resistant cells generated through CRISPR/Cas9 or epigenetic modification, have been reported to improve healthspan-related outcomes. In these models, such cells reduced cellular senescence, supported tissue repair, and slowed age-related functional decline. For example, intravenous administration of senescence-resistant mesenchymal progenitor cells derived from human embryonic stem cells, which are not equivalent to primary MSCs, slowed multi-organ transcriptomic and DNA methylation aging clocks and improved cognitive performance, bone density, and reproductive-tissue biological age in aged cynomolgus monkeys, with no evidence of tumorigenicity [132]. Similarly, EVs from rejuvenated MSCs improved healthspan-related measures in progeroid mice [119].

Together, these advanced engineering approaches highlight the potential of MSCs as a flexible therapeutic platform for age-related diseases and healthspan-relevant outcomes, a potential currently supported primarily by preclinical data [133]. Because many engineering strategies are available, careful comparisons of efficacy and risk will be essential to identify the most suitable options for clinical use [134].

## 5. E-Selectin-Engineered MSCs: A Representative Preclinical Example

**Figure 2:**
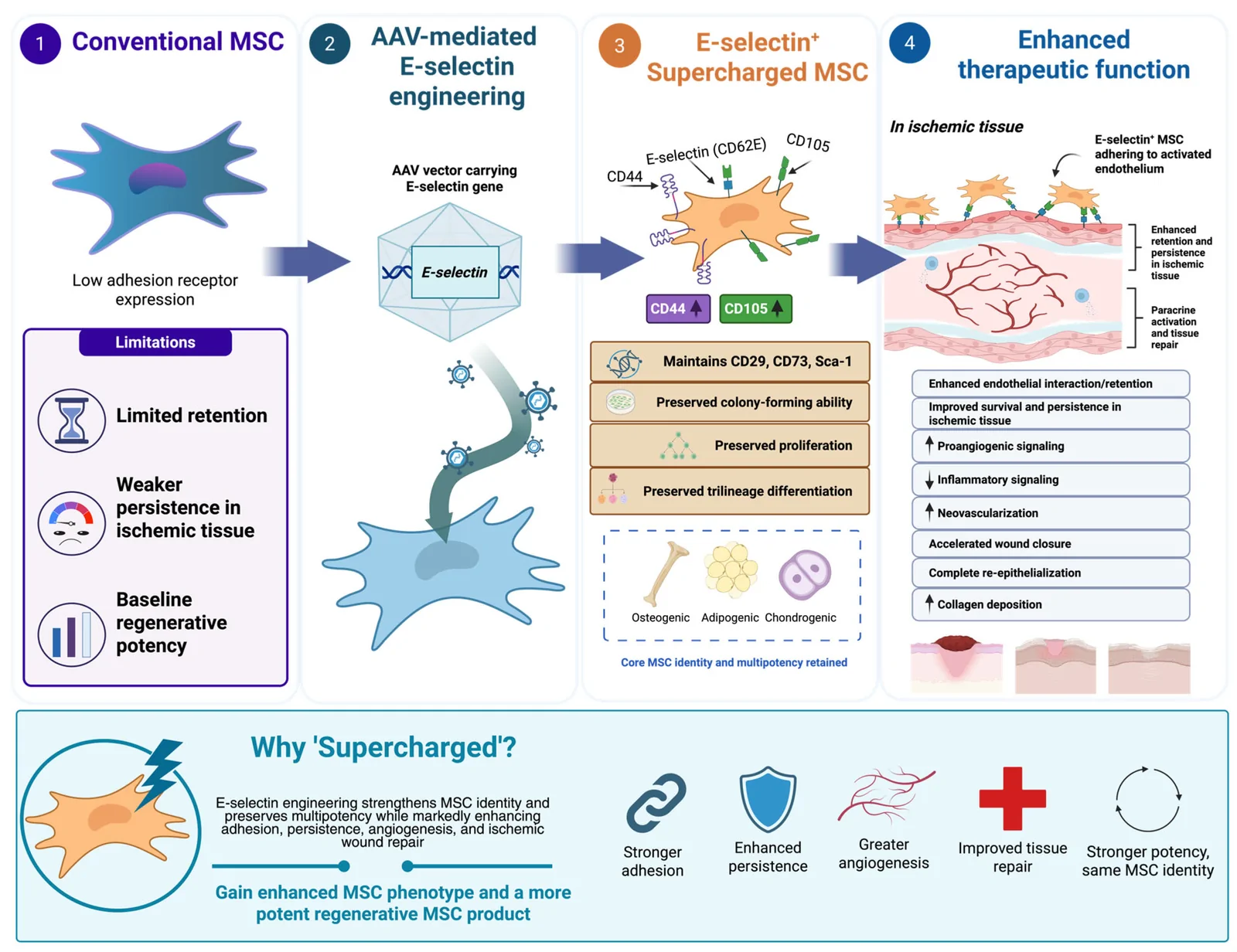
AAV-mediated E-selectin engineering of MSCs. An AAV vector drives surface expression of E-selectin (CD62E), converting conventional MSCs into E-selectin^+^ engineered MSCs that upregulate CD44 and CD105 while retaining core MSC identity and multipotency. In ischemic tissue, these cells adhere to activated endothelium, improving cell retention, angiogenesis, and wound repair. This schematic is conceptual and summarizes preclinical findings only; it should not be interpreted as evidence of clinical efficacy. Created in BioRender. Reme, A. (2026) https://BioRender.com/hsqc8g7.

E-selectin engineering of MSCs is a representative preclinical example of MSC adhesion engineering, not an established therapeutic platform. The supporting evidence remains preclinical and unregistered, and the findings below should be interpreted accordingly. Most of the data originate from one research group, our own, and have not yet been independently replicated. The authors’ patents, licensing agreements, consulting roles, and equity holdings are disclosed in the Conflicts of Interest section. In this strategy, genetic engineering introduces adhesion molecules, such as E-selectin, to enhance MSC homing and persistence at target sites. In this approach, adeno-associated virus (AAV)-mediated transduction is used to induce surface expression of E-selectin in murine bone marrow-derived MSCs. Unmodified MSCs do not express E-selectin; however, after AAV-mediated E-selectin engineering, MSCs upregulate the MSC markers CD44 and CD105 while retaining expression of other standard MSC markers, including CD29, CD73, and Sca-1 [92]. E-selectin-engineered MSCs preserve their fundamental stem/stromal cell properties, including colony-forming capability, proliferative capacity, and trilineage differentiation potential into chondrogenic, osteogenic, and adipogenic lineages [60,92,135]. These marker and differentiation findings still need to be independently replicated to ensure their reliability [60,92]. Figure 2 summarizes this strategy, tracing the transition from conventional MSCs to AAV-mediated E-selectin engineering, culminating in the E-selectin^+^ phenotype and its therapeutic function in preclinical ischemic models.

Functionally, E-selectin-engineered MSCs displayed greater regenerative and proangiogenic activity than control MSCs. In an ischemic wound model, treatment with E-selectin-engineered MSCs significantly accelerated wound closure, promoted complete re-epithelialization, increased collagen deposition, and enhanced vascular density within ischemic wounds [92] (Figure 2). These cells also showed improved survival and persistence in ischemic tissue, suggesting that E-selectin modification may strengthen MSC viability and therapeutic durability in a hostile ischemic microenvironment. Taken together, these preclinical findings indicate that E-selectin engineering may enhance tissue repair and angiogenic capacity in murine models, although these effects await independent confirmation. On this basis, E-selectin-positive MSCs have been described in the primary literature as “supercharged” MSCs [60,92]; for clarity, the neutral term E-selectin-engineered MSCs is used throughout this review.

These cells strongly interact with endothelial ligands in ischemic tissues, thereby enhancing cell retention, promoting neovascularization, and elevating the expression of proangiogenic genes such as Cxcl2 [29,60,81]. In a murine limb ischemia model, E-selectin-engineered MSCs reduced muscle atrophy and enhanced neovascularization [60,136]. In addition to enhancing tissue adhesion, E-selectin also acts as a signaling molecule that increases expression of proangiogenic genes, including Cxcl2, and reduces inflammatory mediators such as TNF, thereby promoting paracrine factor release and cell survival [29,81,136].

By reshaping the molecular microenvironment, this approach may help address key limitations of standard MSC therapies in severe tissue loss, though this remains to be confirmed [137]. Future studies should determine whether E-selectin alters the MSC secretome, recruits host endothelial or immune cells, acts primarily through cell retention or paracrine activation, and whether its benefits persist after transgene expression declines. These studies will help determine whether this strategy should focus primarily on adhesion, paracrine activation, or both [29,92,136].

The long-term effects of E-selectin-engineered MSCs remain unclear, with limited safety and efficacy data available over extended periods. In a mouse model of critical limb ischemia (CLI), E-selectin-engineered MSCs remained localized within the treated tissue, did not engraft in distant organs such as the lungs or liver, and exhibited no evidence of systemic toxicity [60]. These findings align with the overall safety profile of genetically modified MSCs, as Investigational New Drug-enabling toxicology studies have not detected severe adverse events [138]. Clinical meta-analyses indicate that MSC therapy is typically well tolerated, with transient fever being the most frequently observed treatment-related side effect [139,140]. These broader safety data describe unmodified and other genetically modified MSC products and should not be extrapolated to this specific engineered cell. Since most data are derived from short-term or single-dose studies, ongoing monitoring is necessary to assess the long-term stability of the engineered phenotype and any delayed effects, such as tumorigenicity. This is especially important because the therapeutic benefits could decline as transgene expression diminishes [141].

Several limitations specific to AAV-based delivery require careful consideration. Many people already have immunity to common AAV serotypes, which can neutralize the vector and decrease transduction efficiency [142,143]. Transgene expression from AAV is often temporary and inconsistent, making it hard to control the level and duration of E-selectin expression [143]. Manufacturing clinical-grade AAV is complicated, and achieving the necessary vector yields, purity, and batch consistency for scalable production remains a major hurdle. Set against the non-genetic alternatives described in Section 4.3, this approach carries a higher manufacturing and regulatory burden. HCELL glycoengineering achieves selectin-mediated homing transiently and without permanent genetic modification, and has already been evaluated in phase I testing [55], whereas AAV-mediated E-selectin expression remains confined to murine models. It has not been established whether the potential durability benefits of a genetic approach justify the additional burden.

## 6. Manufacturing, Potency, and Regulatory Considerations for Engineered MSC Products

Clinical translation of engineered MSCs depends as much on manufacturing strategy as on biological rationale. During production, MSCs can undergo replicative aging and clonal selection, reducing multipotency and shifting the secretome toward a pro-inflammatory profile even when canonical surface markers remain stable [70]. Each additional engineering step introduces potential variability. Therefore, standardized culture methods and rigorous bioequivalence testing across batches are essential to reduce phenotypic drift and preserve therapeutic potency consistent with the intended mechanism of action [144,145].

Because engineered MSCs may still home inefficiently, effective delivery can require high cell doses. This increases the risk of pulmonary embolism, microthrombi, and vascular occlusion, particularly when infusion parameters are not optimized [68,146,147]. Age-related changes in both donors and recipients may further worsen biodistribution and reduce regenerative effects [68]. Because only a small fraction of administered cells typically engraft, repeated treatments may be required; however, repeated expansion and administration can accelerate MSC aging and reduce signaling capacity [148,149].

Hypoxic preconditioning and optimized delivery strategies may help mitigate these limitations [149]. Nevertheless, donor variability and prolonged ex vivo expansion remain as major obstacles to the production of consistent, high-potency MSC products for reliable clinical outcomes [150]. Potency testing should extend beyond surface-marker analysis and include functional, multi-parameter assays that reflect how engineered cells interact with diseased tissue [151]. Future validation should incorporate real-time monitoring of secretome profiles and cell-surface ligand density to keep batch variability within safe and effective limits [17].

Regulators generally have higher expectations for genetically engineered MSCs than for minimally manipulated products. When viral vectors are used, testing must detect replication-competent viruses and carefully assess the risk of integration to reduce the potential for insertional mutagenesis or tumorigenesis [122,152]. When immortalized or oncogene-transduced lines (such as SV40T- or CRISPR-immortalized cells) are used solely as producer cells for cell-free EV manufacturing, the cells themselves are not administered. Consequently, regulatory attention shifts from the engraftment and tumorigenicity of a live infused cell to the qualification of the acellular product. These platforms should not be assumed to be clinically proven or inherently safe; instead, they require validated clearance and release testing, independent of the immortalization strategy itself [153,154]. Genome-edited products require thorough off-target analysis, integration-site assessment, and clonal-expansion monitoring to ensure genetic stability and safety [122,155].

For surface-engineered MSCs, the main regulatory focus is confirmation of transient surface changes, such as enhanced selectin binding, through functional assays that demonstrate improved homing. These products generally do not require the same genomic integration or off-target analyses as genome-edited cells [62,122,156]. For MSC-derived EV products, oversight focuses on vesicle identity, purity, sterility after filtration, and potency assays that demonstrate consistent secretome activity [121,157,158]. In all cases, product development should include a clear control strategy with defined acceptance ranges for critical quality attributes.

## 7. Strategies to Resolve Limitations of Engineered MSCs

Precise delivery of engineered MSCs to target tissues and organs remains a major challenge. Genetic modifications, such as adding chemokine receptors including CCR7, can guide MSCs to lymphoid tissues for improved targeting [159]. Preconditioning MSCs with cytokines such as IFN-γ can enhance their intrinsic immunosuppressive activity and survival in inflamed environments without requiring extensive genetic manipulation [96,160,161,162]. Using the MSC secretome, particularly EVs, as a cell-free therapeutic platform may avoid risks associated with live-cell transplantation, including tumor formation and poor cell survival [121].

It will also be important to determine the optimal method for delivering human E-selectin, whether through genetic modification or protein engineering, to achieve effective surface expression without compromising MSC function or therapeutic potential. Inducible expression systems provide control over when and where therapeutic proteins are produced. They enable accurate regulation of gene expression, helping minimize off-target effects and reduce the risk of long-term immune responses [163,164]. They also permit fine-tuning of adhesion molecule expression to improve homing while minimizing immune activation [46]. In addition, inducible systems can adjust protein expression levels in response to disease-specific conditions, making MSC therapies more precise.

Optimizing MSC culture conditions can help preserve function and therapeutic potency. Selecting appropriate media, scaffold materials, and bioreactor setups can enhance immunomodulatory and differentiation abilities, along with the expression of engineered surface molecules [152]. Controlling the timing and duration of engineered molecule expression may also reduce immune responses while preserving native MSC benefits. Transient fucosylation of surface proteins, for example, can improve MSC targeting and retention in inflamed tissues and may increase therapeutic efficacy [54,90].

Biomaterial scaffolds and directed delivery systems can localize MSCs to target tissues, reducing systemic dispersion and improving therapeutic outcomes. For instance, injectable hydrogels and polymer-nanoparticle composites can retain engineered MSCs at local treatment sites. By mimicking aspects of the extracellular matrix, these materials support MSC survival and function while reducing genetic risks and potentially improving scalability and immune compatibility [1,48,165]. They can also be loaded with immunosuppressive agents to shape the local microenvironment and prolong MSC retention and activity [23].

Advanced gene-editing tools such as CRISPR/Cas9 enable precise modifications that can improve the therapeutic performance of MSCs. These tools allow targeted insertion or deletion of genes to enhance immunomodulation, survival, or tissue targeting while reducing limitations associated with older viral approaches [159]. Such modifications can create stable, heritable changes that may improve the safety and efficacy of MSC products for clinical use [166]. In addition, engineering MSCs to produce immunosuppressive factors or molecules that inhibit complement and natural killer (NK) cell activity may further reduce immune rejection [167].

## 8. Future Solutions and Clinical Translation for Healthspan-Relevant Applications

Future MSC engineering approaches for healthspan-relevant applications will likely combine strategies to improve targeting, persistence, and resistance to aging. Genetic modifications that increase senescence resistance, combined with biomaterials that support rejuvenation, may prevent cell decline while supporting regeneration and immune function in age-related diseases [77,95,168].

For diabetic wounds, which are characterized by hyperglycemia, oxidative stress, and impaired angiogenesis, MSCs may need to be engineered to release higher levels of angiogenic factors such as vascular endothelial growth factor (VEGF) or angiopoietin-1 (ANG1). Targeted surface modifications or hydrogel encapsulation can help retain these cells locally and improve vascularization, while transient or cell-free formats may help maintain safety [29,155,169,170].

Another promising direction is the development of cell-free therapies using bioengineered MSC-derived EVs. These vesicles can be designed for targeted drug delivery or increased stability in the bloodstream, which may improve therapeutic utility [171]. MSC-derived EVs may avoid the risks of tumorigenesis and immune responses associated with live cell transplantation [23]. For frailty and aging-related conditions, EV-rich products with anti-inflammatory activity may be particularly valuable. MSC-derived EVs are also attractive because they can be produced at scale and characterized more precisely than parental cell products, helping ensure clinical consistency [172]. To further improve manufacturing precision and reduce donor-to-donor variability, researchers have also explored CRISPR-Cas9-generated immortalized cell lines as EV producer cells [117,121].

Immortalized producer cells require a distinct safety assessment. While an immortalized cell line (such as SV40T- or CRISPR-immortalized) can serve as a platform for producing EVs, it differs significantly from an immortalized live-cell product given to patients. In EV production, the producer cell is not infused into patients; therefore, immortalization alone does not automatically make these platforms clinically approved or inherently safe [154]. The final EV product must undergo thorough testing to exclude residual producer cells, cellular debris, oncogenic DNA and RNA, transforming proteins (like SV40T or other immortalizing genes), viral vector components, and other process-related impurities. This testing relies on validated clearance assays, well-defined release specifications, and a consistent manufacturing process to ensure safety before approval for clinical use [153,173].

Although these approaches are promising, clinical translation of healthspan-related benefits remains challenging. Long-term monitoring is essential to ensure safety, particularly with respect to tumorigenic risk [23,174]. The representative clinical trials and translational research involving MSC-based therapies (see Table 2) mainly focus on specific conditions such as critical limb-threatening ischemia, graft-versus-host disease, acute respiratory distress syndrome, and myocardial infarction. Except for frailty, these studies primarily demonstrate MSC safety and potential benefits but do not directly prove an extension of healthspan. Evidence relevant to healthspan remains mostly preclinical, coming from aging and frailty models, as well as studies on senescence, functional decline, inflammaging, and tissue resilience. Building on this combined translational and preclinical knowledge, larger randomized trials are necessary to determine optimal dosing and verify reproducibility. Standardized methods for measuring the secretome and biophysical features of EVs are also required for quality control before broad clinical use [69,175]. Finally, regulatory guidelines that account for age-related recipient biology and batch-to-batch variability in stem cell products will be critical for reducing the risk of treatment failure in older patients [73,76].

## 9. Safety, Study Design, and Clinical Endpoint Considerations

Thorough preclinical testing and standardized manufacturing are necessary to preserve consistency of cell therapy products and reduce risk. New genetic modification tools, including advanced viral vectors and RNA-silencing approaches, are improving MSC engineering and may support the development of broadly available off-the-shelf MSC therapies [176]. The safety of unmodified MSCs has been evaluated extensively in a large and growing body of clinical trials across a wide range of conditions [52,68,177].

To use engineered MSCs safely, consistent manufacturing and rigorous preclinical validation are required to translate enhanced features into healthspan-focused therapies. Study design should account for the limited in vivo persistence of many MSC products, whereas age-related diseases are typically chronic. Therefore, trials should include surrogate biomarkers of biological aging and tissue recovery rather than relying only on mortality-based endpoints. Functional measures such as the six-minute walk test may be useful in selected contexts [178].

Studies should also assess polypharmacy and possible drug-cell interactions that may influence MSC efficacy in older patients with comorbidities [179]. Finally, data-driven analyses of gene-environment interactions may help identify new therapeutic targets and maintain immune regulation during aging. In healthspan-focused trials, primary outcomes should be both clinically meaningful and disease-specific.

These endpoints should include objective measures of physical function and biological aging, such as reductions in the SASP or reversal of age-related immune remodeling, to evaluate therapeutic efficacy in older adults [25,30]. Studies should also track the removal of senescent cells and systemic effects, as the interactions between MSC therapies and existing secretory phenotypes are a key factor influencing therapeutic outcomes [18,94].

## 10. Limitations

No clinical trial involving MSCs or MSC-derived EVs has specifically targeted healthspan extension as a primary goal. In the trials summarized in Table 2, the main focus was usually on safety or disease-focused functions aimed at enhancing healthspan. The most relevant aging-related measure was the change in six-minute walk distance at 180 days in a phase 2b aging-frailty study, which did not achieve its goal, and a phase 3 trial in amyotrophic lateral sclerosis also failed to meet its primary endpoint. As a result, evidence directly related to biological aging remains limited to preclinical studies.

Most of the engineering strategies reviewed have not been directly compared within the same model, making it difficult to rank their relative effectiveness based on existing literature. Specifically, for E-selectin engineering, the supporting data mainly come from our research group and lack independent validation. For MSC-derived EVs, variability in isolation and potency criteria hampers meaningful comparisons across studies.

## 11. Conclusions

Current research and clinical studies suggest that engineered MSCs may benefit conditions such as immune disorders, bone and cartilage injury, and neurological diseases [174]. Nevertheless, unmodified MSCs often show poor tissue targeting, limited persistence, donor-to-donor variability, and functional decline in aged or inflamed environments. Engineering approaches can address these limitations by improving survival, homing, paracrine signaling, resistance to senescence, and manufacturing consistency.

Important challenges remain in making MSC-based therapies safe, effective, and reproducible. These challenges require strong quality control, standardized manufacturing, and risk-mitigation strategies to improve clinical outcomes [180,181,182]. Additional preclinical and clinical studies are needed to understand long-term effects and to optimize the delivery of engineered MSCs, particularly for inflammatory and age-related metabolic disorders [76].

In preclinical models, E-selectin-engineered MSCs illustrate how modifying cell adhesion may enhance tissue repair, whereas HCELL glycoengineering provides a non-genetic strategy by adding an E-selectin ligand to CD44. Both approaches emphasize the importance of cell trafficking in MSC therapy and may inform the design of more effective regenerative treatments. Progress in this field will require integration of engineering, aging biology, potency testing, scalable manufacturing, and well-designed clinical trials.

Safety features such as inducible suicide systems may improve the safety of genetically engineered MSCs by allowing clinicians to control cell growth and persistence after administration [181]. Despite these advances, improved non-viral gene delivery methods, including lipid nanoparticles and polymer polyplexes, are still needed. These methods avoid genomic integration and may be safer than viral vectors, but they must remain efficient in MSCs, which are difficult to transfect [80,183].

Engineered MSCs and MSC-derived EVs may support tissue repair and healthspan-related outcomes, but their success will depend on well-defined mechanisms of action, reliable manufacturing, rigorous safety evaluation, and clinically meaningful endpoints. Inflammation-responsive or doxycycline-inducible promoters may allow cells to release immunomodulatory factors only when needed, reducing off-target effects, immune reactions, and toxicity [164,184]. With improved plasmid designs and cell preparation methods, these advances can make engineered MSC therapies more precise and clinically effective [185,186].

## Figures and Tables

**Table 1:** Human donor studies on age-related changes in MSC abundance and function.

MSC Source	No. of Donors (M:F)	Methods	Age Groups Defined (Years)	Change in MSC Numbers	Change in MSC Functionality	Ref.
Bone marrow	48 (n/a)	CFU-F assay; flow cytometry (ISCT markers); SA-β-gal and ROS assays; RT-qPCR (reverse transcription quantitative PCR); macrophage coculture	Young: <18; Old: >55	CFU-F markedly reduced in old vs. young BM-MSCs (36 vs. 149 colonies per 10^6^ cells)	Increased senescence (SA-β-gal, ROS); impaired suppression of M1 macrophage polarization	[4]
Bone marrow	4 (n/a)	Proliferation assay; osteogenic differentiation; ECM bioactivity assays	Young: 30 and 45; Old: 60 and 80	Reduced proliferation in old BM-MSCs	Impaired osteogenesis; young-donor ECM better supported proliferation and osteogenesis	[5]
Adipose tissue	24 (15:9)	CFU-F assay; SVF yield; proliferation; SA-β-gal; differentiation; migration assay	Child (6–12), young adult (22–27), elderly (60–73)	~70% lower CFU-F frequency in elderly vs. child; SVF yield and proliferation also declined	Reduced adipogenic/osteogenic differentiation and migration; increased senescence	[6]
Bone marrow	67 (34:33)	CFU-F assay; CD45low CD271+ flow cytometry; gene-expression profiling	Young: 19–40; Old: 61–89	MSC number per mL of BM declined with age (CFU-F r = −0.53; flow r = −0.31)	Reduced clonogenic/proliferative capacity; sorted-MSC gene expression largely unchanged	[10]
Adipose tissue	32 (16:16)	CFU-F assay; proliferation; population doubling time; senescence and differentiation assays	Four groups: >20, >50, >60, >70	Decreased CFU-F and proliferation with age; longer doubling time	Reduced osteogenic/chondrogenic differentiation; adipogenic shift; increased senescence	[11]
Bone marrow	175 (95:80)	CFU-F assay; proliferation; tri-lineage differentiation; surface-marker flow cytometry	Adults (ranging from 18 to 93)	Reduced CFU-F number and proliferation with age (significant in females)	Reduced adipogenic differentiation and SSEA-4/CD146/CD274 expression (female donors)	[12]
Adipose tissue	8 (6:2)	Proliferation; population doubling time; differentiation; paracrine ELISA (enzyme-linked immunosorbent assay)	Young: <30; Elderly: >70	Reduced proliferation and longer doubling time with age	Reduced paracrine factors (VEGF, SDF-1α, HGF); poorer osteogenic/adipogenic differentiation	[13]
Adipose tissue (subcutaneous)	31 (0:31)	Proliferation; adipogenic differentiation; senescence markers (p16INK4a, SA-β-gal); mitochondrial ROS	Young: 20–34; Middle aged: 35–49; Old: 65–80	Proliferation declined with advanced age	Reduced adipogenic capacity; increased p16INK4a, SA-β-gal, and ROS	[14]

Studies were selected to illustrate age-associated changes in human donor-derived MSCs, and were required to compare defined younger and older donor groups, to report at least one quantitative measure of MSC number, proliferative capacity, differentiation capacity, or senescence, and to have been published within the last decade. The table is illustrative rather than exhaustive. All included studies reported an age-related decline in MSC number or frequency (e.g., by CFU-F assay), typically accompanied by reduced proliferative and/or differentiation capacity. Some effects were sex-dependent [12] or observed in female-only cohorts [14]. Sex-specific effects were not systematically assessed across the tabulated studies. Most did not stratify outcomes by donor sex, and sex is therefore noted only where the original reports addressed it. Abbreviations: BM-MSC, bone marrow-derived MSC; CFU-F, colony-forming unit-fibroblast; ECM, extracellular matrix; HGF, hepatocyte growth factor; ISCT, International Society for Cell and Gene Therapy; ROS, reactive oxygen species; SA-β-gal, senescence-associated β-galactosidase; SDF-1α, stromal cell-derived factor 1α; SVF, stromal vascular fraction; VEGF, vascular endothelial growth factor.

**Table 2:** Representative clinical trials and translational experience with MSC-based therapies.

Indication (Phase)	Registered Trial (ClinicalTrials.gov); Status; Last Verified	Cell Source, MSC Strategy, and Route	N Enrolled	Primary Endpoint(s)	Key Reported Outcome(s)	Main Translational Message
CLTI/PAD and ischemic ulcers (Phase 2)	Stempeucel-CLI, CLI due to Buerger’s disease (NCT01484574); Completed March 2016; last verified September 2016	Allogeneic BM-MSC; intramuscular into gastrocnemius and peri-ulcer sites	90	Relief of rest pain and reduction in target-limb ulceration, assessed at 6 and 24 months.	At a dose of 2 million cells/kg weight, rest pain and ulcer healing significantly improved in most patients. Amputation-free survival did not differ among the 3 groups at 6-month follow-up [50]	Benefits have been modest or variable, supporting the need for more potent engineered MSCs
Diabetic Foot Ulcers and PAD (Phase 2)	PDA-002 (cenplacel), diabetic foot ulcer ± PAD (NCT02264288); Terminated early for business reasons; last verified May 2026	Human placenta-derived stromal-like cells; intramuscular	159	Proportion of participants achieving complete wound closure within 3 months, with sustained closure maintained for an additional 4 weeks.	32% of subjects with complete wound closure in the treatment group vs. 22% in the placebo group [51]	MSCs are promising, but poor survival and weak retention in hostile wound tissue limit efficacy
Immune and inflammatory diseases, incl. GVHD (Phase 3)	Remestemcel-L (Prochymal), steroid-refractory acute GVHD (NCT00366145) Completed May 2009; last verified February 2022	Allogeneic BM-MSC; intravenous	260	Durable complete response (DCR), defined as complete resolution of aGVHD symptoms for any period of at least 28 days after beginning treatment	Remestemcel-L did not meet the primary endpoint of greater DCR in the intent-to-treat population. Post hoc analyses showed higher day-28 overall response in children and high-risk aGVHD and higher DCR and overall response with liver involvement [52]	MSCs can suppress inflammation, but patient response is inconsistent
Bone/cartilage injury and degenerative osteoarthritis (Phase 2b)	Autologous Adipose Tissue derived MSCs (Joinstem), knee osteoarthritis (NCT02658344); Completed Dec 2016; last verified June 2019	Autologous adipose-derived MSCs; single intra-articular injection	24	Pain, stiffness, and physical function of the knee measured by the Western Ontario and McMaster Universities (WOMAC) at 6 months	WOMAC improved significantly at 6 months in the MSC arm with no significant change in controls; no serious adverse events. On MRI, the cartilage defect was unchanged in the MSC arm but increased in controls [53]	MSCs may improve symptoms, but durable structural regeneration remains uncertain
Osteoporosis/bone targeting (Phase 1)	Autologous fucosylated BM-MSC for osteoporosis (NCT02566655); Completed May 2018; last verified November 2019	Autologous fucosylated (HCELL/CD44 glycoengineered) BM-MSC; intravenous	10	Rate of serious and non-serious adverse events related to the procedure within 24 months	Preliminary results revealed no related adverse effects, no new osteoporotic fractures, and decreased pain from analogue pain scale score (EVA) in 3/4 patients [54,55]	Surface engineering can improve MSC homing without permanent genetic modification
Neurological and neurodegenerative diseases (Phase 3)	NurOwn (MSC-NTF, BCT-002-US), ALS (NCT03280056); Completed September 2020; last verified February 2024	Autologous, BM-MSCs secreting neurotrophic factors; intrathecal	196	Proportion with ≥1.25 point/month improvement in post-treatment slope vs. pretreatment slope in ALSFRS-R score at 28 weeks following 1st injection	Significant improvements in cerebrospinal biomarkers of neuroinflammation, neurodegeneration, and neurotrophic factor support were observed with MSC-NTF. Primary endpoint and key secondary endpoints were not met [56]	MSCs may provide trophic and anti-inflammatory effects, but the phase 3 trial did not demonstrate efficacy; targeting and potency remain barriers
Frailty/aging-related decline (Phase 2b)	Lomecel-B (laromestrocel), aging frailty (NCT03169231) Completed Sep 2021; last verified March 2022	Allogeneic BM-MSC; peripheral intravenous	148	Change from baseline in 6 Minute Walk Test (6MWT) compared to placebo at 6 months post infusion	Primary endpoint was numerically improved but did not reach statistical significance; however, a dose–response analysis showed a statistically significant relationship of increasing dosage to increased change in 6MWT distance at 6 months [57].	This ceiling of unmodified allogeneic BM-MSCs, motivates engineering strategies (potency licensing, homing, persistence) over simply increasing cell numbers.
Cardiovascular ischemic disease (Phase 1/2)	POSEIDON, chronic ischemic cardiomyopathy (NCT01087996); Completed October 2012; last verified May 2015	Autologous vs. allogeneic BM-MSC, transendocardial injection	30	30-day post catheterization incidence of predefined treatment-emergent serious adverse events (SAEs)	One patient in each group had a treatment-emergent SAE (hospitalization for heart failure) within 30 days, with favorable effects on functional capacity, quality of life, and ventricular remodeling. Autologous but not allogeneic MSC therapy was associated with an improvement in 6 min walk test [58].	Allogeneic and autologous cells were comparably safe, but only autologous cells improved functional capacity, suggesting that donor-derived potency, not delivery, limits unmodified MSC products.
Respiratory inflammatory injury/ARDS (Phase 2a)	START, moderate to severe ARDS (NCT02097641); Completed Feb 2018; last verified March 2019	Allogeneic BM-MSC; intravenous	60	Numbers of Patients Occurred Pre-specified Infusion Associated Events Occurring Within 6 h of Study Infusion	No patient experienced any of the associated adverse events. One patient in the MSC group had an unrelated death within 24 h. Viability of MSCs ranged from 36% to 85%. Study not powered for efficacy [59].	Safety was established, but the viability range across administered doses underscores the need for potency and viability release criteria before efficacy can be meaningfully tested
Ischemic Wounds in a Murine Model (Preclinical)	Not a registered trial (preclinical)	AAV-mediated E-selectin engineered murine BM-MSC; intramuscular injection into ischemic hindlimb	44 murine models	Preclinical reperfusion of hind limb ischemia	Increased level of reperfusion in ischemic hind limb treated with both types of MSCs but effect was significantly more potent with E-selectin-engineered MSCs [60].	E-selectin engineering may enhance tissue repair and angiogenic capacity in murine models, though these effects await independent replication
Peripheral nerve injury and Diabetic Peripheral Neuropathy (Phase 1/2)	Allogeneic BM-MSC; diabetic peripheral neuropathy (NCT02387749); Completed December 2016; last verified January 2021	Allogenic BM-MSCs; intravenous	10	Measurement of bFGF and VEGF via ELISA, and Change in Nerve Conduction velocity, latency, and amplitude	Improved blood sugar levels and nerve function 90 days after the transfusion and increased levels of growth factors (bFGF and VEGF) [61]	Higher bFGF and VEGF levels with improved nerve conduction, provide a mechanistic correlate for the observed benefit. However, with only 10 patients and no control arm, the association remains hypothesis-generating.

Trial phase is as registered. Registry status and last-verified dates were checked against ClinicalTrials.gov. Abbreviations: 6MWT, 6 min walk test; aGVHD, acute graft-versus-host disease; ALS, amyotrophic lateral sclerosis; ALSFRS-R, ALS Functional Rating Scale-Revised; ARDS, acute respiratory distress syndrome; bFGF, basic fibroblast growth factor; BM-MSC, bone marrow-derived MSC; CLI, critical limb ischemia; CLTI, chronic limb-threatening ischemia; DCR, durable complete response; ELISA, enzyme-linked immunosorbent assay; GVHD, graft-versus-host disease; HCELL, hematopoietic cell E-/L-selectin ligand; MRI, magnetic resonance imaging; MSC, mesenchymal stem cell; MSC-NTF, MSC-derived neurotrophic factor-secreting cell; PAD, peripheral artery disease; SAE, serious adverse event; VEGF, vascular endothelial growth factor; WOMAC, Western Ontario and McMaster Universities Osteoarthritis Index.

## Data Availability

No new data were created or analyzed in this study. Data sharing is not applicable to this article.

## Abbreviations

The following abbreviations are used in this manuscript:

AAV	Adeno-associated virus
ALS	Amyotrophic lateral sclerosis
AMPK	AMP-activated protein kinase
ANG1	Angiopoietin-1
ARDS	Acute respiratory distress syndrome
ATMP	Advanced therapy medicinal product
BDNF	Brain-derived neurotrophic factor
BM-MSC	Bone marrow-derived mesenchymal stem cell
Cas9	CRISPR-associated protein 9
CFU-F	Colony-forming unit–fibroblast
CLI	Critical limb ischemia
CLTI	Chronic limb-threatening ischemia
CRISPR	Clustered regularly interspaced short palindromic repeats
CXCR1	C-X-C chemokine receptor type 1
CXCR4	C-X-C chemokine receptor type 4
CXCR7	C-X-C chemokine receptor type 7
DNA	Deoxyribonucleic acid
ECM	Extracellular matrix
ELISA	Enzyme-linked immunosorbent assay
EV/EVs	Extracellular vesicle(s)
GMP	Good manufacturing practice
GVHD	Graft-versus-host disease
HCELL	Hematopoietic cell E-/L-selectin ligand
HGF	Hepatocyte growth factor
IDO	Indoleamine 2,3-dioxygenase
IFN-β	Interferon beta
IFN-γ	Interferon gamma
IL-10	Interleukin-10
iPSC	Induced pluripotent stem cell
ISCT	International Society for Cell and Gene Therapy
ISEV	International Society for Extracellular Vesicles
miRNA	MicroRNA
MISEV	Minimal Information for Studies of Extracellular Vesicles
MPC	Mesenchymal progenitor cell
MSC/MSCs	Mesenchymal stem cell(s)
MSC-derived EVs	MSC-derived extracellular vesicles
MSC-NTF	Mesenchymal stem cell-derived neurotrophic factor (cells)
NAD+/NADH	Nicotinamide adenine dinucleotide
NK	Natural killer (cell)
PAD	Peripheral artery disease
RNA	Ribonucleic acid
ROS	Reactive oxygen species
RT-qPCR	Reverse transcription quantitative PCR
SA-β-gal	Senescence-associated β-galactosidase
SASP	Senescence-associated secretory phenotype
SDF-1α	Stromal cell-derived factor 1α
SEC	Size-exclusion chromatography
SV40T	SV40 large T antigen
SVF	Stromal vascular fraction
TFF	Tangential flow filtration
TGF-α	Transforming growth factor alpha
TNF/TNF-α	Tumor necrosis factor (alpha)
VCAM-1	Vascular cell adhesion molecule 1
VEGF	Vascular endothelial growth factor
